# Proteasome 26S Subunit, non-ATPase 3 (PSMD3) Regulates Breast Cancer by Stabilizing HER2 from Degradation

**DOI:** 10.3390/cancers11040527

**Published:** 2019-04-12

**Authors:** Abdulfattah Salah Fararjeh, Li-Ching Chen, Yuan-Soon Ho, Tzu-Chun Cheng, Yun-Ru Liu, Hang-Lung Chang, Hui-Wen Chang, Chih-Hsiung Wu, Shih-Hsin Tu

**Affiliations:** 1 PhD Program for Cancer Molecular Biology and Drug Discovery, College of Medical Science and Technology, Taipei Medical University and Academia Sinica, Taipei 110, Taiwan; d621104004@tmu.edu.tw; 2 TMU Research Center of Cancer Translational Medicine, Taipei Medical University, Taipei 110, Taiwan; d117094003@tmu.edu.tw (L.-C.C.); hoyuansn@tmu.edu.tw (Y.-S.H.); 3 Division of Breast Surgery, Department of Surgery, Taipei Medical University Hospital, Taipei 110, Taiwan; 4 Taipei Cancer Center, Taipei Medical University, Taipei 110, Taiwan; 5 Department of Medical Laboratory, Taipei Medical University Hospital, Taipei 110, Taiwan; g160090005@tmu.edu.tw; 6 School of Medical Laboratory Science and Biotechnology, College of Medical Science and Technology, Taipei Medical University, Taipei 110, Taiwan; d119096007@tmu.edu.tw; 7 Joint Biobank, Office of Human Research, Taipei Medical University, Taipei 110, Taiwan; d90444002@tmu.edu.tw; 8 Department of Surgery, EnChu Kong Hospital, New Taipei City 237, Taiwan; changhl0321@gmail.com (H.-L.C.); chwu@tmu.edu.tw (C.-H.W.); 9 Department of Surgery, School of Medicine, College of Medicine, Taipei Medical University, Taipei 110, Taiwan

**Keywords:** proteasome 26S subunit, non-ATPase 3 (PSMD3), human epidermal growth factor receptor 2 (HER2), breast cancer, ubiquitination

## Abstract

It is well-known that human epidermal growth factor receptor 2 (HER2) is critical for breast cancer (BC) development and progression. Several studies have revealed the role of the ubiquitin/proteasome system (UPS) in cancer. In this study, we investigated the expression level of Proteasome 26S subunit, non-ATPase 3 (PSMD3) in BC using BC cell lines, human BC tissue samples, Oncomine, and TCGA databases and studied the PSMD3-HER2 protein interaction. PSMD3 was upregulated in BC, particularly in the HER2+ subtype. PSMD3 immunostaining was detected in the cytoplasm and nucleus of BC tumor tissues. Strong interaction between PSMD3 and HER2 at the protein level was observed. Knockdown of PSMD3 significantly impaired the stability of HER2, inhibited BC cell proliferation and colony formation, and induced cell apoptosis. Ubiquitination process was strongly enhanced after knockdown of PSMD3 in association with decreased HER2 level. Accumulation and Localization of LAMP-1 in the cell membrane with decreased HER2 immunostaining was observed after knockdown of PSMD3. High expression level of PSMD3 was associated with HER2 expression (*p* < 0.001), tumor size (*p* < 0.001), and clinical stage (*p* = 0.036). High expression level of PSMD3 predicted a short overall survival (OS), particularly for HER2+. Overall, we provide a novel function for PSMD3 in stabilizing HER2 from degradation in HER2+ BC, which suggests that PSMD3 is a novel target for HER2+ BC.

## 1. Introduction

Breast cancer (BC) is considered the most common malignancy affecting women worldwide and the second leading cause of death in women after lung cancer [1]. BC has been classified based on variations in gene expression into four groups: basal-like group, *ERBB2*-overexpressing group, estrogen receptor (ER) group, and normal-like group, and these groups are highly related to prognosis and clinical outcome prediction [2,3]. Human epidermal growth factor receptor 2 (HER2) is a transmembrane glycoprotein located on chromosome 17 (ch17q12) that controls BC carcinogenesis and tumorigenesis [4,5]. It is known that HER2 overexpression accounts for 15–30% of BC patients with highly associated metastasis and poor clinical prognosis [6]. Trastuzumab (Herceptin) is a monoclonal antibody that has been proven to be effective to treat HER2+ BC, and treatment with Herceptin after adjuvant therapy is effective on OS [7,8,9]. However, resistance to Trastuzumab in BC is a common problem despite the significant improvement in OS [10,11].

The ubiquitin (Ub)/proteasome system (UPS) is responsible for degrading 80–90% of all proteins to maintain efficient cell processes in eukaryotic cells [12]. Most cytosolic and nuclear proteins, including misfolded and short-lived proteins, are targeted by UPS [13]. Proteins targeted for degradation are initially tagged with ubiquitin, a small 76 amino acid protein, at one or more lysine residues as mono-ubiquitination or as a sequence of covalently attached ubiquitin molecules (poly-ubiquitination) [12,14]. Ubiquitination is a multistep process mediated by several ubiquitination enzymes as follows: E1 (ubiquitin-activating enzymes), E2 (ubiquitin-conjugating enzymes), and E3 (ubiquitin ligation enzymes) [15,16]. Polyubiquitinated proteins are recognized and translocated into the inner part of the proteasome 26S through the non-catalytic 19S subunits for proteolytic activity by an ATP-dependent process [17]. The conjugation of ubiquitin is a reversal step through the cleavage of the isopeptide bond at the C-terminal of ubiquitin by Deubiquitinase enzymes (DUBs) [18]. In the human genome, more than 98 DUBs have been identified and categorized into six families that have roles in various cellular functions [18,19]. Three DUBs, PSMD14 [20], USP14 [21], and UCHL5 [22], have been identified in the 19S proteasome complex.

UPS has crucial roles in regulating several biological processes, such as cell cycle processes, cell growth, and migration, in addition to controlling DNA damage, transcription, and apoptosis [23,24]. Thus far, altering UPS functions or changes in expression or mutation in one or more of the UPS components is associated with the etiology of numerous human diseases, in particular cancer [25,26].

In BC, a recent study revealed the role of PSMD2 (proteasome 26S subunit, non-ATPase 2) in controlling cell proliferation and the cell cycle of BC cells by modulating p21 and p27 turnover through the ubiquitination process and deubiquitinating by USP14 [27]. PSMD2 was also reported as a potential target for lung adenocarcinoma. The results demonstrated that knockdown of PSMD2 led to decreased proteasome activity and induced apoptosis through the induction of p21 [28]. Increased expression of cNrf2 via the reciprocal talk between cNrf2 and PSMD4 (proteasome 26S subunit, non-ATPase 4) promoted colorectal cancer with more aggressive tumors in in vitro and in vivo models. Moreover, when a proteasome inhibitor was applied, the metastasis ability was completely suppressed [29]. Recent research further suggested the role of PSMD4 in colorectal cancer chemoresistance via the cNrf2-mediated signaling cascade [30]. The association of the proteasome with cell membrane receptors, such as HER2, in BC is not fully understood. Marx et al. [31] demonstrated that bortezomib, a 20S proteasome inhibitor, reduced proteasome activity, inhibited cell growth, and enhanced ER and HER2 degradation by inducing apoptosis, as indicated by poly ADP ribose polymerase (PARP). However, the molecular basis for the interaction between the proteasome and cell receptors, such as HER2, remains unclear. Proteasome 26S subunit, non-ATPase 3 (PSMD3), is a part of the 19S lid complex. PSMD3 is located at ch17q21 and encodes a protein with a molecular weight (Mw) of 60 kDa. PSMD3 is expressed in most of the tissues according to the RNA-Seq database [32]. It has been reported that PSMD3 genotypes are associated with WBC and neutrophil counts and have interactions with dietary fatty acids and carbohydrates on glucose-related traits [33,34]. A recent study demonstrated that HER2 expression is strongly correlated with its surrounding genes, including PSMD3, which is co-expressed with HER2 in BC [35]. Co-silencing of PSMD3 and HER2 gave an additive effect for inhibiting the viability and cell proliferation of the tumor cells rather than a single treatment in the KPL4 cell line [36]. A brief report revealed that PSMD3 expression was significantly associated with HER2 and detected in 23% of HER2+ tumors [37].

In this study, PSMD3 was upregulated in BC and associated with HER2+, with strong interaction between PSMD3 and HER2 at the protein level. Our results provide a novel function for PSMD3 in stabilizing HER2 from degradation. Knockdown of PSMD3 in several HER2+ BC cells destabilized HER2 and enhanced HER2 degradation.

## 2. Results

### 2.1. PSMD3 Expression Is Coupled with HER2 Expression

**Figure 1:**
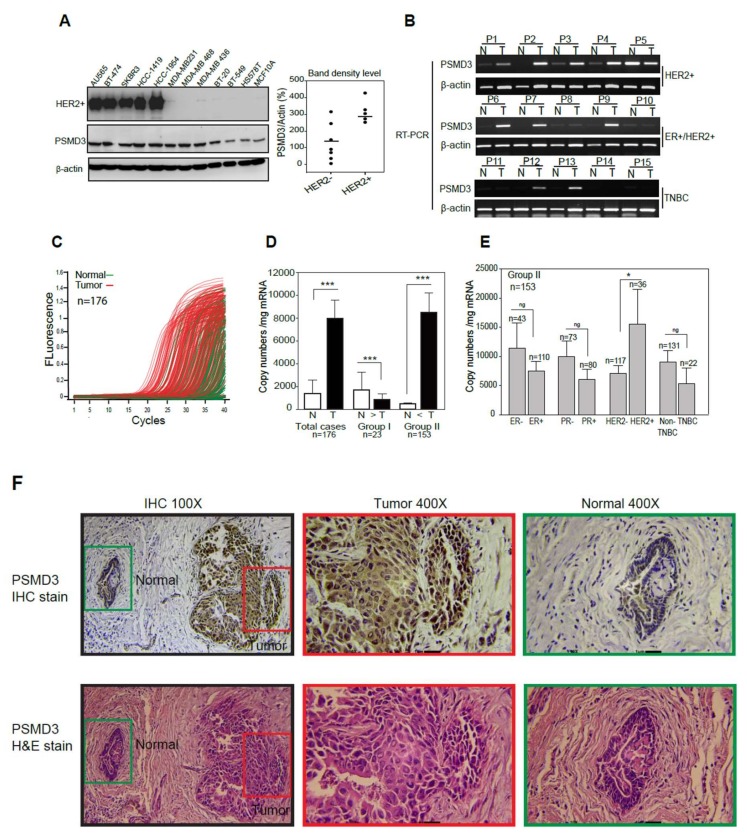
Detection of Proteasome 26S subunit, non-ATPase 3 (PSMD3) expression level in normal and malignant breast cancer (BC) cell lines and human BC tissues. (**A**) PSMD3 expression at protein level in Human epidermal growth factor receptor 2 (HER2) positive versus HER2 negative were detected by Western blot; normal (MCF10A) and cancerous human breast cell lines (Au565, BT-474, SKBR3, HCC-1419, HCC-1954, MDA-MB231, MDA-MB 468, MDA-MB 436, B-20, BT-549, H578T). β-actin served as internal control. PSMD3 bands densities were normalize to β-actin. (**B**) Detection of PSMD3 expression at mRNA level in 15 pairs normal versus tumor breast tissue samples were detected by reverse transcriptase (RT)- polymerase chain reaction (PCR). β-actin served as internal control. (**C**) The PSMD3 mRNA expression profiles of paired human breast tumor (red lines) and normal (green lines) tissues (*n* = 176) were detected by real-time PCR. (**D**) Comparison of PSMD3 mRNA expression between normal (N) and tumor (T) pairs (*n* = 176), copy number (mg /mRNA). PSMD3 mRNA expression levels in 23 patients’ samples in which PSMD3 mRNA expression was higher in normal tissue than in tumor tissue (Group 1) versus 153 patient samples in which expression was higher in tumor tissue than normal tissue (Group 2). Error bars indicate the 95% confidence interval. Data was analyzed with 2-sided paired *t*-test (*** *p* <0.001). (**E**) Analysis of PSMD3 copy number levels between BC subtypes, including (Estrogen Receptor; ER− vs. ER−, Progesterone receptor; PR− vs. PR+, HER2− vs. HER2+, and Triple Negative Breast Cancer; TNBC vs. Non-TNBC). Error bars indicate the 95% confidence interval. Data was analyzed with independent t-test (* *p* < 0.05). (**F**) Representative images for PSMD3 Immunohistochemistry (IHC) immunostaining Tumor area versus normal area (Upper panel) (100× and 400×) and hematoxylin and eosin staining (lower panel) (100× and 400×).

A panel of HER2+ and HER2- BC cell lines and MCF 10A normal breast cells were used to examine the protein expression of PSMD3. As shown in (Figure 1A), PSMD3 was upregulated in all HER2+ cells with relatively greater percentages than HER2- cells. We further confirmed PSMD3 expression at the mRNA level using pairs of breast tissue samples, normal (N) versus tumor (T), by reverse transcription polymerase chain reaction (RT-PCR); PSMD3 was upregulated in 7 out of 10 HER2+ tumor tissues (Figure 1B), compared to the relative normal tissues.

Next, we examined the level of PSMD3 mRNA in pairs of tumor and adjacent normal breast tissue samples using quantitative real-time PCR (*n* = 176) (Figure 1C). PCR amplification curves generated from tumor samples were shifted forward (red lines) relative to curves generated from normal tissues (green lines), indicating relatively higher PSMD3 expression in tumor tissues. The samples were divided into two groups according to PSMD3 copy number (CN) [38,39] (Figure 1D). Group 1 included patients with PSMD3 at a higher level in normal (N) tissues than in tumor (T) tissues (N > T, 23/176, 13%). Group 2 included patients with a higher PSMD3 level in tumors tissues than in normal tissues (T > N, 153/176, 87%); the results indicated the upregulation of PSMD3 in tumor tissues. Importantly, PSMD3 was highly expressed and associated with the HER2+ subtype (*n* = 36, *p* = 0.050 *). No such significant association was observed in ER, PR, or TNBC patients (Figure 1E). To confirm the expression pattern of PSMD3 in BC, Immunohistochemistry (IHC) was performed on BC tissues. Cytoplasmic and nuclear immunostaining of PSMD3 was markedly detected in tumor tissues compared with normal tissues, which have low PSMD3 immunostaining. Representative IHC staining images for PSMD3 are shown in Figure 1F. Altogether, these results suggested that PSMD3 was upregulated in BC cell lines and tissues compared to normal cells and normal tissues and co-expression with HER2 in BC.

### 2.2. PSMD3 Expression Is Associated with HER2+ Patients, According to the Oncomine and TCGA Databases

To confirm our results, we used the Oncomine and TCGA databases [40] to analyze PSMD3 expression with HER2 in multiple cohort studies. PSMD3 was elevated in patients with HER2+ BC compared to HER2- BC, according to Gluck and Zhao BC statistics (Figure S1A). Furthermore, the correlation between the expression of PSMD3 and HER2 in BC was also very high according to the Zhang BC statistics (*n* = 313) using a heat map (Figure S1B). PSMD3 gene expression level from the TCGA-BRCA database was downloaded (*n* = 522), and the correlation of PSMD3 with different BC subtypes was analyzed accordingly. Patients were divided into two groups based on the mean level of PSMD3: group I, PSMD3 low (*n* = 91); and group II, PSMD3 high (*n* = 431). As shown in Figure S1C, the highest level of PSMD3 was detected in tumor tissues with HER2+ expression. Importantly, these results are consistent with our findings, which showed that HER2 and PSMD3 are co-expressed and associated in BC.

### 2.3. Direct Interaction Between PSMD3 and HER2 in HER2+ BC Cell Lines

**Figure 2:**
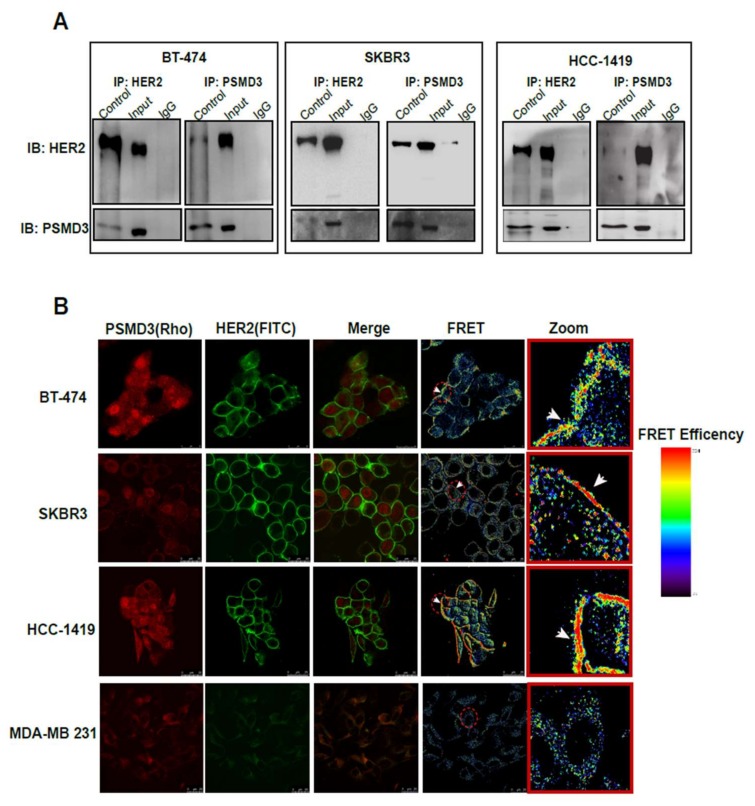
Direct interaction between HER2 and PSMD3 in BT474, SKBR3, and HCC-1419. (**A**) Total cell lysates from HER2+ cells were incubated with either PSMD3 or HER2 antibodies at 4 °C overnight. Immunoprecipitation (IP) were subjected to western blot analysis with anti-PSMD3 or anti-HER2 antibodies. (**B**) BT-474, SKBR3, HCC-1419, and MDA-MB231 were stained with PSMD3 and HER2 antibodies at 4 °C overnight. Secondary antibodies conjugated with Rhodamine and FITC against anti-PSMD3 and anti-HER2, respectively, were incubated for an hour at room temperature. Confocal Fluorescence resonance energy transfer (FRET) assay was used to analyze the protein–protein interaction. Scale Bar is 25 µM.

To investigate the association of PSMD3 with HER2 at protein level, Immunoprecipitation (IP) and Fluorescence Resonance Energy Transfer (FRET) assays were performed. As shown in Figure 2A, three different HER2+ cell lines (BT-474, SKBR3, HCC1419) were used to detect PSMD3-HER2 protein interactions. After immunoprecipitation of HER2, PSMD3 was detected in all of the HER2+ cells. The same result was observed for the IP of PSMD3, while HER2 was detected in all tested cells. Next, a FRET assay by confocal microscopy was performed to confirm the direct interaction between PSMD3 and HER2. FRET relies on the distance-dependent transfer of energy from a donor molecule to an acceptor molecule. After immunostaining BT-474, SKBR3, and HCC1419 cells with primary antibodies anti-HER2 and anti-PSMD3, secondary antibodies conjugated with Rhodamine and Fluorescein isothiocyanate (FITC) were applied. As shown in the images (Figure 2B), a strong interaction between PSMD3 and HER2 was detected in all HER2+ cells. However, there was no interaction observed in MDA-MB 231, a HER2- cell between PSMD3 and HER2. Overall, these results indicate the presence of a direct interaction between HER2 and PSMD3 in HER2+ cells.

### 2.4. PSMD3 Stabilizes HER2 at Protein Level

**Figure 3:**
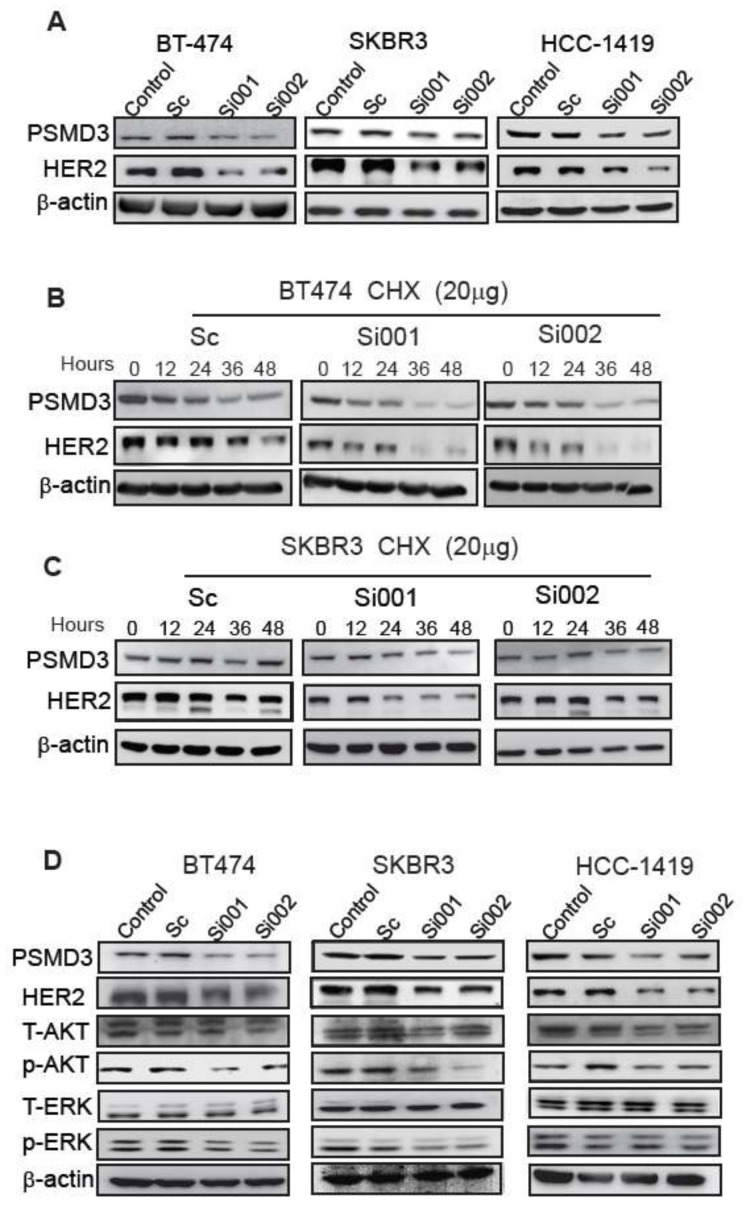
PSMD3 expression is essential to maintain HER2 stability. (**A**) Twenty-four hours after transfection of BT474, SKBR3, and HCC-1419 with either scramble (Sc), PSMD3-Si001, or PSMD3-Si002. The cell lysates were collected and analyzed by western blot to detect the PSMD3 and HER2 protein levels. (**B**) Scramble, PSMD3-si001, or PSMD3-Si002 for BT474 and SKBR3 were treated with Cycloheximide (CHX) at 20 µg. The cells lysates were obtained at the indicated time points and analyzed by western blot. (**C**) BT474, SKBR3, and HCC-1419 were treated with Scramble, PSMD3-Si001, or PSMD3-Si002 for 24 h and the cells were lysed. The samples were resolved by SDS-PAGE and subjected to western blot analyses with indicated antibodies. (**D**) BT474, SKBR3 and HCC-1419 were transfected with Scramble, PSMD3-Si001or PSMD3-Si002 for 24 h, then the cells were lysed and the proteins were extracted. The samples were analyzed by western blot to detect the PSMD3, HER2, T-AKT, p-AKT, T-ERK and p-ERK, β-actin served as internal control.

A previous study reported the role of the proteasome in regulating the ERBB2 and ERBB2 pathway in BC [31]. However, the exact correlation between the proteasome and HER2 is still unclear. This prompted us to investigate whether the strong interaction between PSMD3 and HER2 could stabilize the HER2 protein. Several HER2+ cells with two different PSMD3-siRNA and two controls were used to study whether the loss of PSMD3 function would affect the HER2 stability. As shown in (Figure 3A), 24 h after transfection of BT-474, SKBR3, and HCC-1419 cells with scrambled PSMD3-Si001 or PSMD3-Si002 plasmids, the total HER2 (185 kDa) in siRNA-transfected cells was significantly reduced compared to un-transfected cells or cells transfected with the scrambled siRNA.

To further confirm the effect of PSMD3 on HER2 stability, a Cycloheximide (CHX) chase assay was performed. After 24 h of transfection, BT-474 and SKBR3 were treated with 20 µg/mL CHX, and cells were harvested at 0 h, 12 h, 24 h, 36 h, and 48 h. Knockdown of PSMD3 significantly impaired the stability of HER2 in the two cell lines BT-474 and SKBR3, whereas the scrambled siRNA had no such effect (Figure 3B). The two PSMD3-siRNAs-expressing cell lines exhibited significant knockdown of PSMD3 levels compared with the control.

### 2.5. PSMD3 Silencing Inhibits Cell Proliferation and Induces Cellular Apoptosis

We next asked whether knockdown of PSMD3 could also affect HER2 signaling pathways. Previously, treatment of BT-474 and SKBR3 cells with 5 nM bortezomib for 48 h inhibited the phosphorylation of AKT but not the ERK signaling pathway [31]. We found that knockdown of PSMD3 in BT-474, SKBR3, and HCC-1419 cells led to decrease of the total form of HER2 and inhibition the main HER signaling pathways, p-AKT and p-ERK; however, no significant effect was observed for the un-transfected cells or cells transfected with the scrambled siRNA (Figure 3D). These data indicated that PSMD3 regulates HER2 stability and inhibits the main HER2 signaling pathways (AKT and ERK).

**Figure 4:**
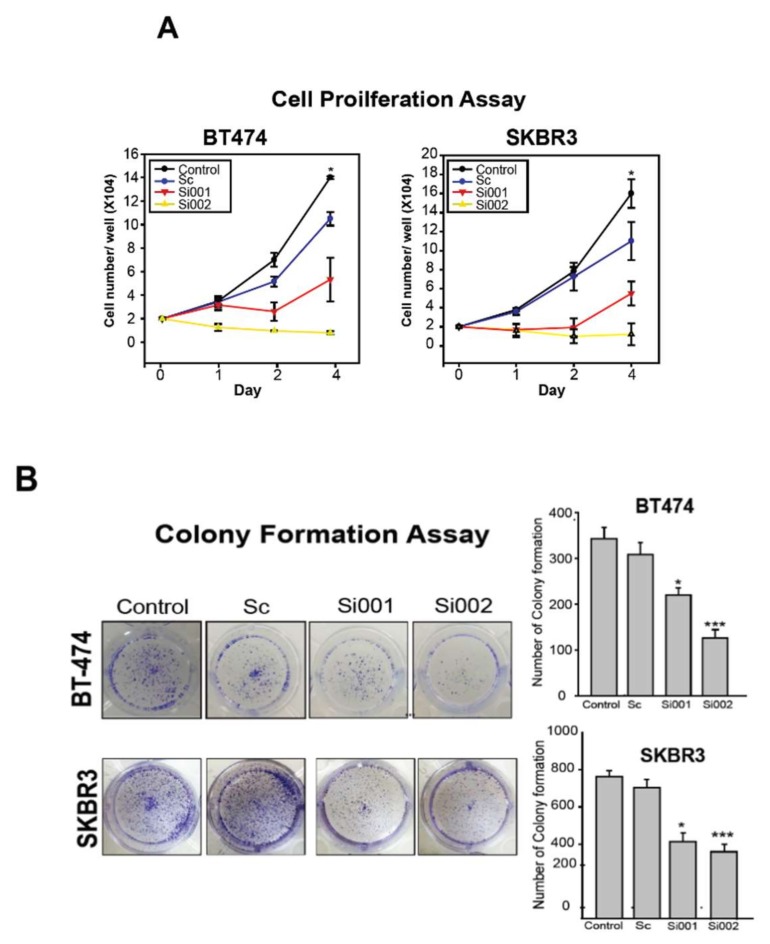

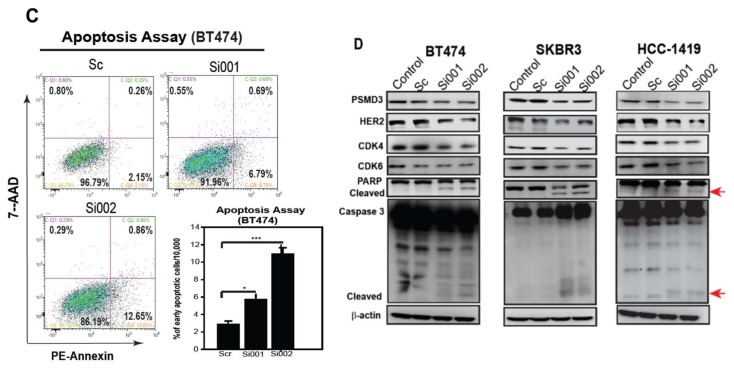
Silencing of PSMD3 inhibits cell proliferation and induces cell apoptosis. (**A**) Cell proliferation assay for BT474 and SKBR3. After transfection the cells with Scramble (Sc), PSMD3-Si001, or PSMD3-Si002, cells (2X10^4^) were seeded in a 24-well dish and cells number were counted at day 1, day 2, and day 4. Data are presented as the mean ± S.D * p < 0.05. (**B**) BT474 and SKBR3 were seeded in a 12-well culture plate after being treated with the indicated plasmids. After 10 days, the cells were fixed and stained with crystal violet. Data are presented as the mean ± S.D (* *p* < 0.05 *** *p* < 0.001). (**C**) Annexin V, cell apoptosis assay by flow cytometry. Twenty-four hours after treating the cells with the indicated plasmids, cells were collected and washed two times with PBS and stained with PE-Annexin or 7AAD. Data are presented as mean ± S.D (* *p* < 0.05 *** *p* < 0.001). (**D**) The expression of cell cycle and apoptotic related markers for the HER2 positive cell lines were detected by western blot (PSMD3, HER2, CDK4, CDK6, PARP, and caspase3). β-actin served as internal control.

Cell proliferation assay by counting the number of cells was used to investigate whether PSMD3 knockdown attenuated cell proliferation in BT-474 and SKBR3 BC cells. As shown in Figure 4A, PSMD3-Si001 or PSMD3-Si002 significantly attenuated cell proliferation; however, no differences were observed in the controls. To evaluate the abilities of BC cells to form colonies, a colony formation assay was performed. BT-474 and SKBR3 were transfected with the indicated plasmids and seeded at low cell density with normal medium in 12-well plates for 10 days. The colonies were fixed and stained. The un-transfected cells or cells transfected with the scrambled siRNA yielded normal colonies with strong and high density staining. However, PSMD3-Si001 or PSMD3-Si002 colonies for BT-474 and SKBR3 were notably inhibited (Figure 4B).

Inhibited cell proliferation in BT474 and SKBR3 after loss of PSMD3 function prompted us to analyze the apoptosis status of the cells. An apoptosis assay (PE-Annexin V/7AAD) was performed. After 24 h of transfection with scrambled siRNA, PSMD3-Si001, or PSMD3-Si002, BT-474 cells were collected, washed, and stained with PE-Annexin or 7-AAD according to the manufacturer’s protocols, and the samples were analyzed by flow cytometry. Only GFP plasmid transfected cells were selected for flow cytometry analysis. Cells exposed to UV light were used as a positive control. Our data confirmed that the populations of BT-474 cells were increased in PE-Annexin-positive and 7-AAD-negative cells after silencing PSMD3 with PSMD3-Si001 or PSMD3-Si002 compared to the control. The early apoptosis indices for the scrambled siRNA, PSMD3-Si001, and PSMD3-Si002 were 2.15%, 6.79%, and 12.65%, respectively (Figure 4C).

To identify the mechanism by which the attenuation of cell growth and induction of cell apoptosis after knockdown of PSMD3, a western blot analysis of cell cycle- and apoptosis-related signal proteins (CDK4, CDK6, PARP, cleaved caspase-3) was performed (Figure 4D). Our data indicated significant downregulation of CDK4 and CDK6 and marked activation of PARP and caspase-3 after silencing of PSMD3 in BT-474, SKBR3, and HCC-1419 cells. However, there were no differences for the un-transfected cells or cells transfected with scrambled siRNA.

### 2.6. Silencing of PSMD3 Enhanced Ubiquitination and Lysosomal Process in Correlation with Decrease HER2 Level

**Figure 5:**
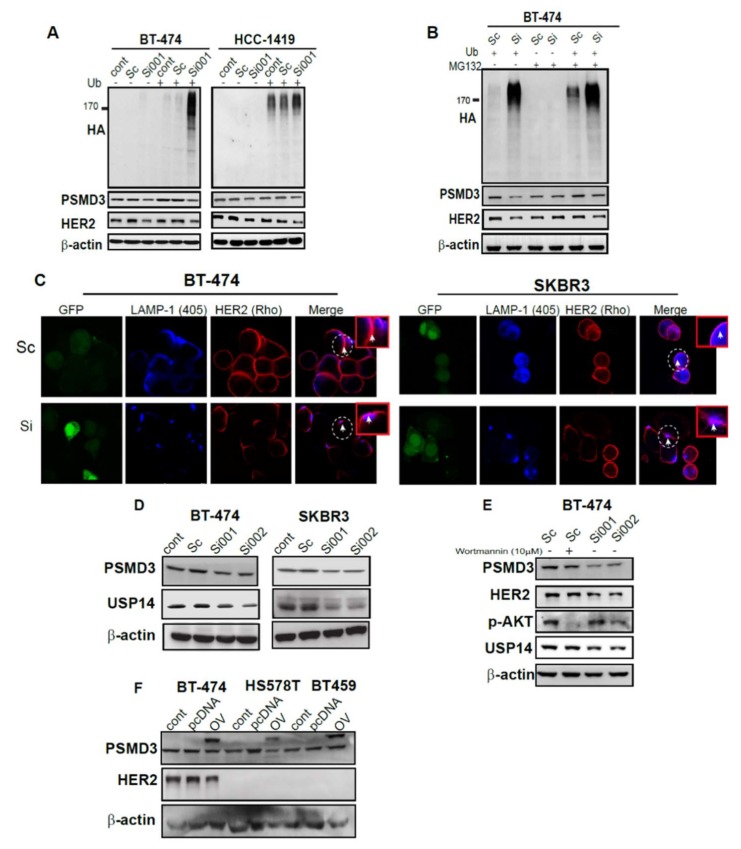
Enhancing the ubiquitination and lysosomal degradation pathway through silencing of PSMD3. (**A**) BT-474 and HCC-1419 control. Scramble and PSMD3-Si001 were co-transfected with or without ubiquitin plasmid for 24 h and then lysed. The cell lysates were incubated with antibodies against Ubiquitin (HA), HER2, and PSMD3. β-actin served as a control. (**B**) BT-474 was treated with Scramble and PSMD3-Si001 were co-transfected with or without ubiquitin plasmid for 24 h and then treated with or without MG132 at a concentration of 5µM for 8 hours and lysed. The cell lysate was incubated with the indicated antibodies. (**C**) Confocal microscope pictures of SKBR3 and BT474 treated with scramble or PSMD3 for 24 h and incubated with HER2 and LAMP-1 lysosomal marker antibodies and showing HER2-LAMP-1 co-localization. Scale bar is 25 µM. (**D**) B-474, SKBR3 cell lysates were incubated with PSMD3 and USP14 antibodies. Western blot analysis to detect PSMD3 and USP14 protein levels was used. β-actin served as a control. (**E**) B-474 transfected with Scramble, PSMD3 si001, or PSMD3 si002 for 24 h. Control cells were treated with Wortmanine inhibitor for 3 hours. Western blot analysis to detect PSMD3, HER2, p-AKT, and USP14 protein levels was used. β-actin served as a control. (**F**) Overexpression of PSMD3 in BT474, HS578T, and BT459. Western blot data showing the expression of PSMD3, HER2, and β-actin; * indicates the PSMD3 conjugated with Yellow fluorescent protein (YFP) fusion protein, where the band shifted upward.

We next investigated the status of ubiquitination after knockdown of PSDM3. A ubiquitination assay was used. After 24 h of co-transfection of scrambled siRNA or PSMD3-Si001 with or without the HA-ubiquitin plasmid, BT-474 and HCC-1419 cells were collected, and western blotting was used to determine the ubiquitination status. Increased ubiquitination at HER2 position was more strongly enhanced and more evident after knockdown of PSMD3 in the two HER2+ cell lines (BT-474 and HCC1-419). However, no such effect was observed for the control or the scrambled siRNA-treated cells (Figure 5A). Furthermore, treatment with MG132 alone, a 26S proteasome inhibitor, did not enhanced the ubiquitination and no changes were observed on PSMD3 or HER2 protein level. However, treatment with MG132 and co-transfection of the cells with HA-ubiquitin and PSMD3 Si plasmids resulted in a decrease in HER2 protein levels, as shown by the intensive level of ubiquitination at HER2 position in BT-474 cells, while no such changes were observed for the cells treated with scrambled siRNA (Figure 5B). These results suggest that the ubiquitination process was more actively processed in PSMD3-silenced cells and HER2 level was decreased in cells treated with MG132 and PSMD3 Si001 and HA-tag ubiqutin plasmids.

To gain more insight about the HER2 degradation process, 24 h after treatment of BT474 and SKBR3 cells with either scrambled siRNA or PSMD3-Si were incubated with HER2 and lysosomal marker (LAMP-1) antibodies for immunofluorescence (IF) staining. Confocal microscopy was used to detect HER2 and lysosomal co-localization after silencing of PSMD3 (Figure 5C). Unsurprisingly, the scrambled siRNA -treated cells showed a clear plasma membrane localization of HER2 in BT474 and SKBR3 cells. A decrease of HER2 IF staining was observed after knockdown of PSMD3. Furthermore, accumulation of LAMP-1 in the cell membrane with co-localizing HER2 was observed, as indicated in the white circle in BT474 and SKBR3 (Figure 5C), indicating the decay of HER2 by a lysosomal-dependent manner. 

DUBs enzyme acts in trimming the ubiqutinated proteins. We hypothesized that intensive ubiquitination should be associated with loss of DUBs function. USP14 was reported to be associated with PSMD2 in breast cancer and it is considering as a major regulator of proteasome by ubiquitin chain disassembly [27]. This finding prompted us to test whether USP14 would be affected after PSMD3 knockdown. As shown in Figure 5D, PSMD3 silencing in BT474 and SKBR3 led to decreased protein level of UPS14. A previous study has reported that phosphorylation and activation of USP14 was regulated by AKT. We used Wortmannin (PI3K inhibitor) to test whether PSMD3 or USP14 protein level is regulated by AKT pathway. Wortmannin successfully inhibited the phosphorylation of AKT without any significant changes for PSMD3 or USP14 protein level. However, knockdown of PSMD3 led to decreased HER2 and USP14 protein levels, while no such change was observed for the cells treated with scrambled siRNA (Figure 5E).

### 2.7. PSMD3 Is Dependent on Proteasome

Next, we asked whether overexpression of PSMD3 acts independently of proteasome and enhances HER2 protein level. After 24 h of transfection of BT-474 as a HER2+ and HS578T and BT459 as HER2- cell lines with the PSMD3-overexpression plasmid (pcDNA3.1+/fusion protein YFP), Western blot was used to detect the HER2 protein level. As shown in Figure 5F, the exogenous PSMD3 overexpression band shifted up (PSMD3 linked with YFP) with no enhancement to HER2 protein level in BT474, HS578T, or BT459. In addition, the exogenous PSMD3 band has no role in activation in either p-AKT or p-ERK in HER2+ or HER2- cell lines (Figure S2). These results indicated that PSMD3 is dependent on proteasome.

### 2.8. Correlation of PSMD3 Expression with Clinicopathological Features in BC

**Figure 6:**
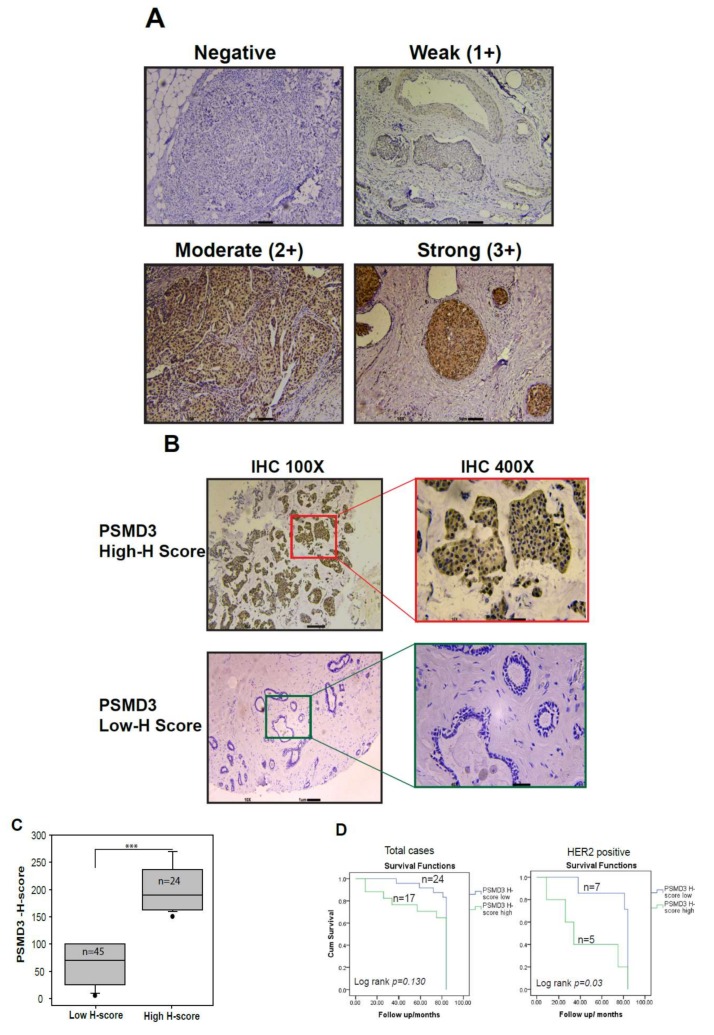

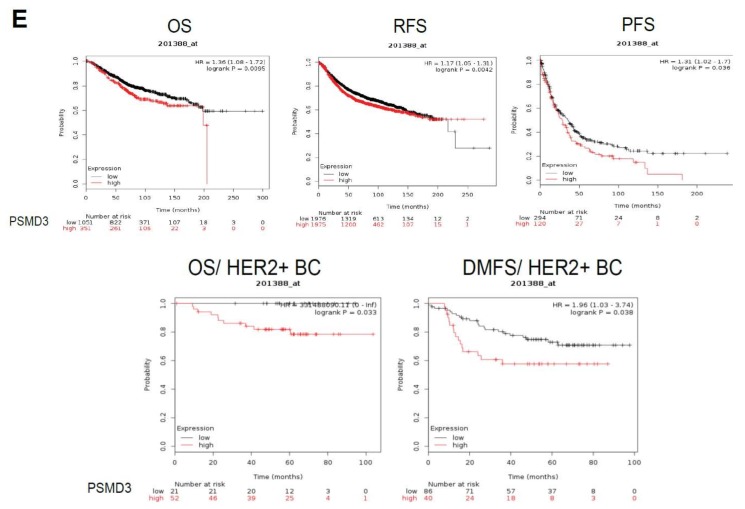
Detection of PSMD3 expression by IHC staining and association of PSMD3 with clinical overall survival. (**A**) Representative images for PSMD3 IHC scoring system. The scoring system was determined as Negative (0), Weak (1+), Moderate (2+), and Strong (3+) based on PSMD3 staining intensity. Scale bar is 1 µM (10×). (**B**) Representative images for low H-score (normal breast cells) versus high H-Score (tumor breast cells). Note the cytoplasmic and nuclear staining in tumor cells (original magnification 100× and ×400). (**C**) Histological analysis of PSMD3 expression in BC patients based on H-score value (*p* < 0.001 ***). (**D**) Kaplan–Meier survival curves of breast cancer patients. A comparison of low- and high-PSMD3 H-score in HER2+ versus total cases. The difference between the survival curves was calculated using the log-rank test. PSMD3 expression level was calculated using PSMD3 protein H-score. (**E**) Kaplan–Meier Plotter survival curves of breast cancer (BC) patients. Comparison of low- and high-PSMD3 mRNA level in BC Overall survival (OS), Relapse-free survival (RFS), and progression-free survival (PFS) and in HER2+ BC; (OS) and distant metastasis-free survival (DMFS). The difference between the survival curves was calculated using the log-rank test.

To further investigate the relationship of PSMD3 expression at the mRNA and protein levels with clinical pathological parameters in BC, the TCGA-PSMD3 gene expression database, Formalin Fixed Paraffin embedded (FFPE) sections (*n* = 10), and tissue microarray (59) cases were used in this study. We evaluated the immunostaining of PSMD3 in FFPE and TMA specimens (Figure 6A) using the IHC scoring system, and the scoring system was determined as Negative (0), Weak (1+), Moderate (2+), and Strong (3+) based on the PSMD3 staining intensity. Representative images for the cases that were considered as having a high H-score compared with those with a low H-score are shown in Figure 6B. The patients were divided into two groups, low PSMD3 (low H-score) or high PSMD3 (high H-score). Out of 69 cases, 10 cases were normal tissues, 25 (35%) had a higher PSMD3 H-score, and 44 cases (65%) had a low H-score; most of the normal tissues (*n* = 8/10) had a low H-score. Regarding clinical outcomes, no significant difference was observed for the OS in patients with high versus low PSMD3 according to Kaplan–Meier survival curves. Interestingly, the HER2+ group with a higher PSMD3 level predicted worse overall survival (log rank *p* = 0.03) (Figure 6C). The Kaplan–Meier plotter database was used to analyze the clinical outcomes for BC patients with high versus low levels of PSMD3 using mRNA levels. High PSMD3 expression showed poor Overall survival (OS), Relapse-free survival (RFS), and progression-free survival (PFS) in BC and poor OS and distant metastasis-free survival (DMFS) in HER2+/BC compared with low PSMD3 expression (Figure 6E), which is consistent with our results. According to the TCGA database, Table 1 summarizes the association of PSMD3 with BC molecular subtype (HER2 Status, ER status, and TNBC status), node status, tumor size, and stage of the disease. High PSMD3 expression was significantly associated with HER2+ BC subtypes *(p* < 0.001), tumor size (*p* < 0.001), and clinical stage (*p* = 0.036). No associations were found with ER status, TNBC status, or node status according to the TCGA database.

Our results were based on the protein H-score for PSMD3. No significant differences between BC subtypes (HER2, ER, or TNBC patients) or for age, clinical stage, grade, or node were found in both groups (Table S1). However, Univariate and Multivariate Cox regression analyses showed that only PSMD3 was an independent predictor for OS, particularly in HER2+ patients (Tables S2 and S3).

## 3. Discussions

The roles of the proteasome in cancer has been intensively studied. Recently, several studies revealed the critical role of UPS components, the lid subunits (19S), or the 20S subunits in several types of cancers [27,28,29,30,41]. In this study, for the first time, we investigated the PSMD3 expression level in BC using BC cell lines and paired human tissue samples from BC patients. In addition, we performed several functional analyses to analyze the PSMD3-HER2 protein–protein interaction. PSMD3 was upregulated in BC cell lines and tumor tissues were compared to normal breast cells and tissues. The sixth HER2+ cell lines, HER2+ tumor tissues, and patients with HER2+ from the Oncomine and TCGA databases showed significantly higher levels of PSMD3 compared to normal breast tissue or to other BC subtypes. Interestingly, strong protein–protein interaction between PSMD3-HER2 was observed by IP and FRET assays.

HER2 is located at chr17q12 and in agreement with a previous study on the chr17q copy number (CN) patterns for HER2 and HER2-related genes. PSMD3s locate at ch17q21 and is considered as one of the close genes surrounding HER2 that exhibited high copy number (CN) in parallel with HER2 [35]. Due to the positive correlation between HER2 and PSMD3 in BC, loss of PSMD3 function analyses in several HER2+ cells was performed. Indeed, silencing of PSMD3 led to destabilization of total HER2 (185 kDa), and HER2 expression was significantly reduced by more than 60% compared to the controls by using two different PSMD3-siRNA.

The main role of the proteasome is to control cell death and apoptosis [42]. Several studies have revealed that inhibiting proteasomal subunits leads to the induction of apoptotic cell death in cancer cells. PSMD4 has been shown to correlate with PARP to induce cell apoptosis [41]. Silencing of PSMD2 regulated cell cycle and apoptosis by modulating p21 and p27 in BC [27]. Our results revealed that downregulation of HER2 by silencing of PSMD3 not only provides a significant inhibition of cell proliferation but also results in activation of cellular apoptosis (measured by Annexin V and activated PARP and caspase-3) in HER2+ cells through inhibition of the main HER2 signaling pathways (ERK/AKT).

In the present study, we showed that transfecting BT-474 and HCC1419 cells with the ubiquitin plasmid had no effect on the ubiquitination process with normal level of HER2. Interestingly, co-transfection of BT474 and HCC1419 with ubiquitin plasmid and PSMD3 Si led to a strongly enhanced ubiquitination process and decrease of the total level of HER2. We treated HER2+ BC cells with MG132, a proteasome inhibitor, with or without silencing PSMD3 in the presence or absence of ubiquitin plasmid. We found that MG132 with co-transfection of the cells with ubiquitin and PSMD3 Si plasmids had an effect on enhancing HER2 degradation by non-proteasome process. Collectively, we uncovered the role of PSMD3 in stabilizing HER2.

Cell receptor targeting usually sensitizes the receptors for internalization by a process called receptor-mediated endocytosis (RME), with an association with lysosomal degradation [43]. A number of reports have been published on the degradation of HER2 by a lysosomal-dependent process [44,45]. A previous study demonstrated the use of an anti-HER2 aptamer grafted onto nanostars that mediated HER2 endocytosis and lysosomal degradation [46]. This finding prompted us to investigate whether silencing PSMD3 enhances HER2 degradation by lysosomal pathway. Interestingly, we found that LAMP-1, a lysosomal marker, was accumulated at the cell membrane with decreased HER2 immunostaining compared with the preserved form of HER2 at the cell membrane in the control group, suggesting that HER2 is degraded by the lysosomal pathway.

DUBs are considered an important part of the UPS system, and the fundamental role of DUBs is, specifically, to disassemble ubiquitin from target proteins. DUBs contribute to the regulation of various cellular processes, such as preserving proteins from degradation [47], regulating UPS or lysosomal-dependent protein degradation [48], and cell cycle or apoptosis [49]. Among the DUBs that have been identified in the human genome, USP14 has been implicated in several types of cancers, including brain [50], ovarian [51], and liver [52] cancers. A recent study revealed the enhancement of ubiquitination and the degradation of the androgen receptor (AR) in prostate cancer as a result of inhibiting USP14 [53,54]. PSMD3 is not considered a DUB enzyme and does not belong to the ATPase subunits. Interestingly, we found that the protein level of USP14 decreased after knockdown of PSMD3 in HER2+ cells, and thus could inhibit trimming of the ubiquitin from HER2. According to Xu et al., phosphorylated and activated USP14 is regulated by the AKT pathway [55]. We demonstrated that inhibition of the AKT pathway did not affect the PSMD3 or USP14 protein level. However, knockdown of PSMD3 led to decreased USP14 protein level.

An overexpression assay was used to overexpress PSMD3 in BC cells. Unexpectedly, the results indicated that PSMD3 has no any oncogenic role in enhancing HER protein level, which indicating that PSMD3 is a proteasome dependent protein. 

To the best of our knowledge, we show here for the first time the clinicopathological correlation of PSMD3 with BC using FFPE and TMA analyses and using Oncomine and TCGA databases. Patients with high PSMD3 expression had worse OS, RFS, and PFS for BC and OS, and DMFS for HER2+BC, suggesting the use of PSMD3 as an unfavorable prognostic factor for BC patients. Our results also revealed that patients who were HER2+ had higher PSMD3 levels and shorter OS. Similar results were obtained by univariate and multivariate Cox regression analyses. Unexpectedly, we did not find any significant correlation between low versus high PSMD3 in either the clinical stage, grade, tumor, or node status when we used protein H-score results or between BC molecular subtypes because of the low case numbers that were included in this study. However, according to the TCGA database, high PSMD3 levels have significant differences with tumor size, stage of the disease, and importantly, HER2 status. To our knowledge, this is a novel finding that would be worth validating by using a large series of BC patients based on PSMD3 protein expression and further confirming the correlation between PSMD3 levels and clinicopathological parameters of BC.

## 4. Materials and Methods

### 4.1. Clinical Patient Samples

All pairs of human breast tumor samples and adjacent normal epithelial tissues (*n* = 176) were obtained from anonymous donors according to a protocol approved by the Institutional Review Board (TMU-JIRB, 20170119). In this study, all participants provided approval with their written consent forms. The Research Ethics Committee of the Taipei Medical University Hospital approved the study and consent procedures. Histological inspection showed that more than 80% of the cells in each tumor tissue were cancer cells.

### 4.2. Cell lines and Cell Culture

All of the BC cell lines, including AU-565, BT-474, SKBR3, HCC1419, HCC1954, MDA-MB-231, MDA-MB-468, MDA-MB-436, BT-20, BT-549, Hs 578T, and the normal breast cell line MCF 10A were purchased from American Type Culture Collection (ATCC, Manassas, VA, USA) and were used in this study. For BC cell lines, cells were maintained in 1:1 DMEM/Ham’s F12 (Thermo Fisher Scientific, Waltham, MA, USA), supplemented with 10% fetal bovine serum (FBS) and antibiotic (5 mg/mL penicillin, 5 mg/mL streptomycin, and 10 mg/mL neomycin). For MCF 10A, cells were maintained in complete medium (1:1 DMEM/Ham’s F12, 10 mg/mL insulin, 0.5 mg/mL hydrocortisol, 20 ng/mL epidermal growth factor, 10% fetal bovine serum (FBS), and antibiotic (5 mg/mL penicillin, 5 mg/mL streptomycin, and 10 mg/mL neomycin)). All the cells were maintained in a 37 °C incubator with 5% CO_2_.

### 4.3. RNA Isolation, Conventional PCR, and Real-Time Quantitative PCR

Total RNA from BC cell lines and human breast tissue samples (tumor and normal) was extracted using Trizol reagent (Invitrogen, Carlsbad, CA, USA) according to the manufacturer’s protocol. PCR primers included the PSMD3-specific forward 5′-ACGTGAAACAGCTAGAGAAA-3′ and reverse 5′-CATCATGAAGATGACCACGA-3′ and the β-actin-specific forward 5′-TGTACGTTGCTATCCAGGCT-3′ and reverse 5′-CTCCTTAATGTCACGCACGA-3′ primers. A Light Cycler thermocycler (Roche Molecular Biochemical, Mannheim, Germany) was used for real-time quantitative PCR. PSMD3 mRNA fluorescence intensity was measured and normalized to GUS expression using the built-in software (Roche Light Cycler, version 4).

### 4.4. Oncomine, TCGA and Kaplan-Meier Plotter Data Set Analysis

Data from the Oncomine gene expression array datasets (https://www.oncomine.org/resource/login.html) and data from the TCGA-BC cohort by UCSC (University of California at Santa Cruz, CA, USA) Cancer Genomics Browser (https://xenabrowser.net/) were used to analyze the correlation of PSMD3 at the mRNA level with HER2 in BC. Kaplan-Meier plotter database analysis was used to analyze the clinical significance of PSMD3 in BC (http://kmplot.com/analysis/) [56].

### 4.5. Immunohistochemistry (IHC) Staining and Scoring System

To elucidate the clinical significance of PSMD3 expression in correlation with clinicopathological parameters in BC, IHC staining for PSMD3 was performed in FFPE and TMA BC tissues, which included a total of *n* = 69 BC cases. TMA was purchased from Super-Biochips Laboratories (Catalog no: CBA4, Seoul, Korea), and the paraffin samples were constructed from BC tissues obtained from the Taipei Medical University Hospital, Taipei, Taiwan. FFPE BC-tissue samples were sectioned at a thickness of 5 μm and deparaffinzied and dehydrated. For antigen retrieval, the slides were pretreated by heating at 95 °C for 25 min in a retrieval buffer of citric acid (pH = 6.0). The sections were preincubated with 3% H_2_O_2_ for 5 min to quench superoxidase, and then the slides were incubated with rabbit polyclonal antibody raised against PSMD3 (1:150 dilution) in a humidified chamber at 4 °C overnight. The staining was developed according to the Dako REAL EnVision Detection System (Glestrop, Denmark). PSMD3 against normal breast tissues was used as a negative control. Immunostaining of PSMD3 was examined by specialized pathologists. Staining was evaluated based on the intensity and the percentage of positively stained tumor cells. PSMD3-positive cells were defined as tumor cells if the cytoplasm and nuclei were at least weakly stained. The H-score was calculated using the following equation: H-score = *Pi* (*i*+1), where *i* is the intensity of the stained tumor cells (0 to 3+), and *Pi* is the percentage of stained tumor cells for each intensity [38]. The cutoff value was set as 120%, which was the mean of the H-score. Cases that exceeded 120% were considered high PSMD3 expression.

### 4.6. Immunoprecipitation (IP)

Cell lysates (0.8–1 mg) were precleared by adding 10 µL of protein A or G agarose beads for 1 h at 4 °C. After centrifugation at 1200× *g*, the supernatant was incubated with primary antibodies overnight at 4 °C. IgG was used as a negative control. Then, 15 µL of protein A or G agarose beads were added and incubated for 2 h at 4 °C. After centrifugation at 1200× *g*, the pellet was collected and washed twice with cell lysis buffer. Immunoprecipitated proteins were resolved on 12% sodium dodecyl sulfate-polyacrylamide gel electrophoresis (SDS-PAGE) and subsequently analyzed by Western blot.

### 4.7. Immunoblotting

Equal amounts of cellular proteins were resolved by electrophoresis in 12% (SDS-PAGE) and transferred to a nitrocellulose membrane for immunoblotting using specific antibodies. Antibodies against HER2 and PARP were purchased from Santa Cruz Biotechnology (Santa Cruz, CA, USA). Antibodies against AKT and P-AKT were purchased from Cell Signaling Technology, Inc. (Danvers, MA, USA); antibodies against CDK4, CDK6, Caspase 3 and LAMP-1 were purchased from Genetex (Taiwan); antibodies against PSMD3 were purchased from Bethyl Laboratory (Montgomery, AL, USA). The β-actin was used as an internal control and purchased from Sigma (Montgomery, AL, USA). Primary antibodies were incubated at 4 °C overnight, followed by incubation with secondary antibodies (goat anti-mouse or goat anti-rabbit) for an hour at room temperature. The antigen-antibody complexes were detected using the Western Blotting Luminol Reagent Kit (Santa Cruz, CA, USA).

### 4.8. Construction of Plasmids

Scrambled and RNAi sequences for PSMD3 were designed using Oligoengine 2.0 software. The selected sequences were cloned into the pSUPER.retro.neo+gfp vector according to the pSUPER RNAi System protocols (Oligoengine Co., Seattle, WA, USA). For PSMD3 overexpression, a vector that contained the PSMD3 gene (full-length CDC mRNA 1605 bp) was purchased from Applied Biological Materials (ABM) Inc. (British columbia, Canada). The gene was cloned into the pcDNA3.1 + His/myc-YFP fusion protein overexpression vector (Invitrogen, Carlsbad, CA, USA) to generate the pcDNA3.1 + PSMD3 overexpression vector. The pSUPER-siPSMD3, pSUPER-scPSMD3, and pcDNA3.1 + His/myc-YFP PSMD3 overexpression vectors (OV) were transfected by electroporation into BT-474, SKBR3, or HCC1419 cells using a pipette-type microporator MP-100 (Invitrogen, Carlsbad, CA, USA).

### 4.9. Immunofluorescence (IF) Staining and Confocal Microscopy 

Cells were fixed with 4% paraformaldehyde for 10 min at room temperature and permeabilized with 0.1% Triton X-100 in PBS for 5 min at room temperature. Samples were blocked for 30 min in PBS with 2% bovine serum albumin (BSA). Primary antibodies were diluted 1/100 with 1% BSA in PBS and then incubated overnight at 4 °C. Secondary antibodies Fluorescein (FITC) AffiniPure Goat Anti-Mouse IgG (H+L) (Jackson ImmunoResearch, West Grove, PA, USA), Rhodamine (TRITC) AffiniPure Goat Anti-Rabbit IgG (H+L) (Jackson ImmunoResearch), and DyLight™ 405 AffiniPure Goat Anti-Mouse IgG (H+L) (Jackson ImmunoResearch) were diluted 1/50 and incubated for 1 h at room temperature. Coverslips were mounted with VECTASHIELD Antifade Mounting Medium (Vector Laboratories, Burlingame, CA, USA) and imaged by confocal microscopy (Leica, Wetzlar, Germany).

### 4.10. Cell Proliferation and Colony Formation Assays

To measure cell proliferation, BT-474 and SKBR3 cells were seeded in 24-well culture plates with a cell density of 2 × 10^4^ cells/well after transfection with the indicated plasmids. The cell numbers were counted at day 1, day 2, and day 4. For the colony formation assay, after treating BT-474 and SKBR3 cells with the indicated plasmid, the cells were seeded into 12-well plates at 1000 cells per well in triplicate and cultured for 10 days until the cells were grown in colonies. Cells were then fixed with methanol for 10 min and stained with crystal violet. The colonies were counted using Image J software (version 1.8, University of Wisconsin, Madison, WI, USA).

### 4.11. Apoptotic Assay

After 24 h of transfection with the indicated plasmids, the cells were collected in 100 μL cell binding buffer. Then, the cells were stained with PE Annexin V or 7AAD according to the Annexin V Apoptosis Detection Kit (BD Biosciences, Pharmingen, San Diego, CA, USA). The samples were analyzed using flow cytometry (BD Biosciences, Franklin, Lakes, NJ, USA).

### 4.12. Ubiquitination Assay

After 24 h of transfection with the indicated plasmid and HA-ubiquitin plasmid, the cells were collected and lysed. Western blotting was used to analyze the ubiquitination status for the controls and PSMD3 siRNA.

### 4.13. Statistical Analysis

The data were analyzed using SPSS version 18.0 (SPSS, Inc., Chicago, IL, USA). Data analysis was conducted by paired and unpaired independent *t*-tests or chi-squared tests. Cox-regression analysis was used for univariate and multivariate analyses to analyze the clinical prognosis significance in BC. Kaplan–Meier analysis was used to plot survival curves, which were compared by the log-rank test. Statistical significance was defined as * *p* < 0.05 and *** *p* < 0.001.

## 5. Conclusions

**Figure 7:**
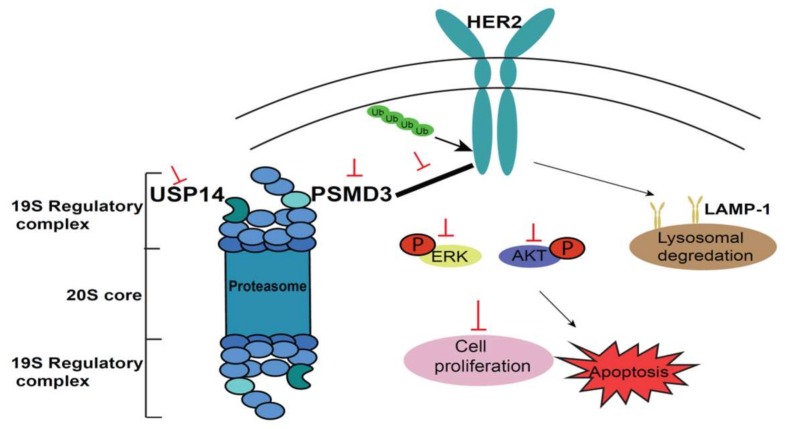
A schematic molecular model for PSMD3 in the stabilization of HER2. USP14 is a DUB enzyme that trims ubiquitin (Ub) from substrates. PSMD3 interacts with HER2, which maintains HER2 and HER2 signaling pathways. Loss of PSMD3 function led to destabilization of HER2 and enhanced ubiquitination and association with decreased HER2 level and lysosomal degradation pathway. The major HER2 signaling pathways (AKT and ERK) were inhibited, leading to the inhibition of cell proliferation and the induction of cellular apoptosis.

We revealed the expression pattern of PSMD3 in BC cell lines and human tissue samples with an association with a clinical prognostic significance. More importantly, this study provided novel insight into the interaction of the proteasome-associated proteins PSMD3 and HER2 and identified that PSMD3 serves as a HER2-stabilizing protein on the cell membrane and could be considered a novel target for the HER2 BC subtype (Figure 7).

## Figures and Tables

**Table 1:** Correlation between PSMD3 gene expression and Clinicopathological parameters of breast cancer using TCGA data (*n* = 1212).

Parameters	Categories	PSMD3 Low mRNA(*n*)	PSMD3 High mRNA(*n*)	*p*-Value
HER2 status	Positive	52	133	0.000 ***
	Negative	164	163	
		NA = 700		
ER status	Positive	151	205	0.874
	Negative	65	91	
		†NA = 700		
TNBC	TNBC	49	49	0.082
	Non-TNBC	167	247	
		†NA = 700		
Tumor size	T1	173	135	0.000 ***
	T2	340	366	
	T3	75	75	
	T4	6	30	
		†NA = 12		
Node	N0	288	273	0.787
	N1	203	213	
	N2	63	69	
	N3	53	50	
Stage	I	120	89	0.036 *
	II	343	348	
	III	130	148	
	IV	13	21	

ER, Estrogen receptor; HER2, human epidermal growth factor2; TNBC, triple-negative BC; † NA, not given. Clinicopathological parameters were assessed using Chi-square analysis. * *p* < 0.05, *** *p* < 0.001. PSMD3 low vs PSMD3 high based on the mean level of PSMD3.

## Supplementary Materials

The following are available online at https://www.mdpi.com/2072-6694/11/4/527/s1, Figure S1: Correlation between PSMD3 and HER2 mRNA expression according to Oncomine and TCGA databases, Figure S2: PSMD3 overexpression and its relation to HER2 and HER2 signaling proteins in BT474 and HS578T cell lines. Table S1: Correlation between PSMD3 H-score and Clinicopathological parameters of breast cancer (*n* = 59), Table S2: Univariate and multivariate analysis of OS in HER2 positive vs total patients according to PSMD3 H-score, Table S3: Univariate and multivariate analysis of overall survival (OS) according to TCGA database.
