# Supplementary material for: Proteasome 26S Subunit, non-ATPase 3 (PSMD3) Regulates Breast Cancer by Stabilizing HER2 from Degradation
Source: Cancers (Basel). 2019 Apr 12;11(4):527. doi: 10.3390/cancers11040527 (PMC6549480; doi:10.3390/cancers11040527)
Supplement: Supplementary file 1 [file cancers-11-00527-s001.pdf]

Supplementary Materials: Proteasome 26S subunit, non-ATPase 3 (PSMD3) regulates breast cancer by stabilizing HER2 from degradation

Abdulfattah Salah Fararjeh, Li-Ching Chen, Yuan-Soon Ho, Tzu-Chun Cheng, Yun-Ru Liu, Hang-Lung Chang, Hui-Wen Chang, Chih-Hsiung Wu and Shih-Hsin Tu

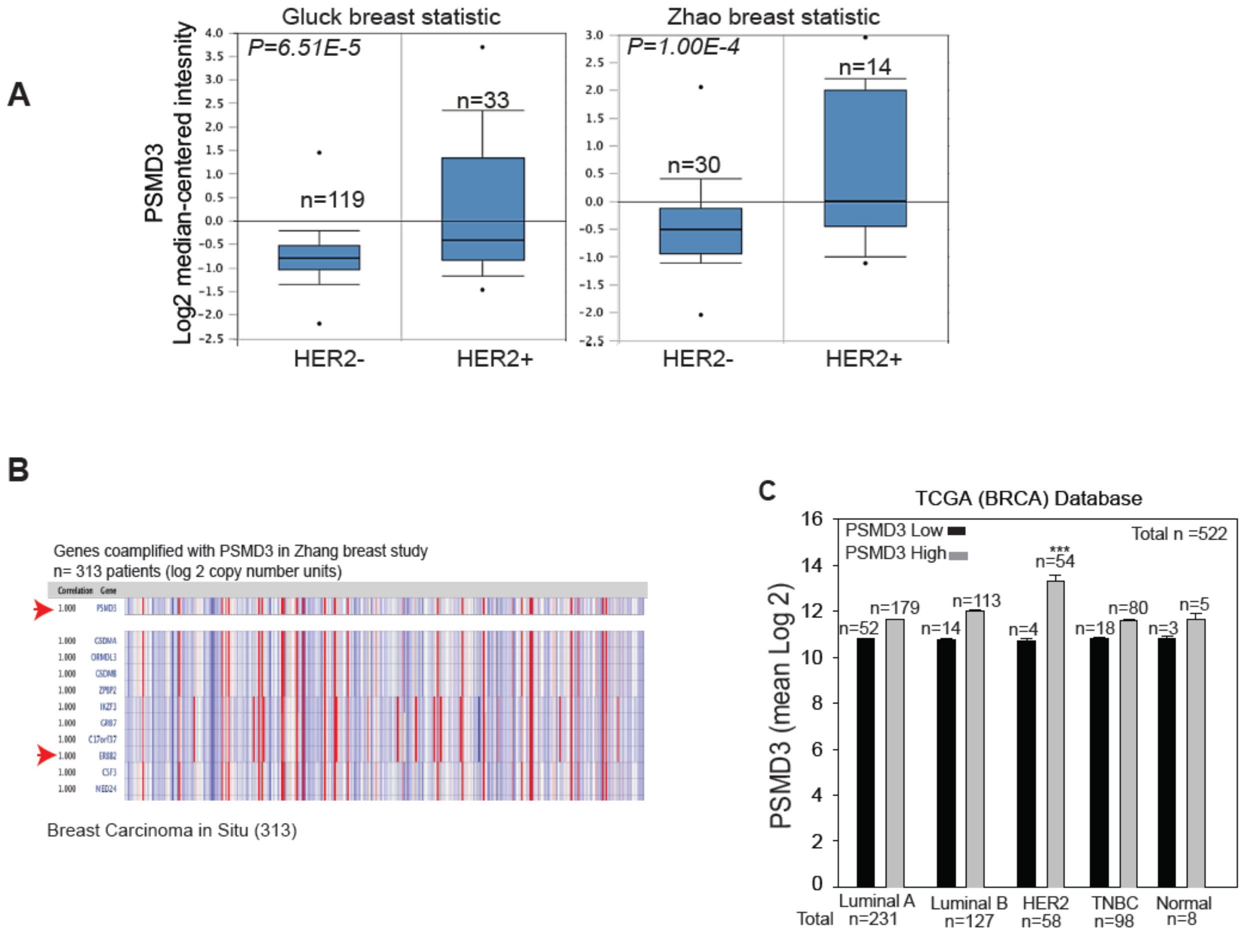

Figure S1. Correlation between PSMD3 and HER2 mRNA expression according to Oncomine (**A** and **B**) and TCGA (**C**) databases.

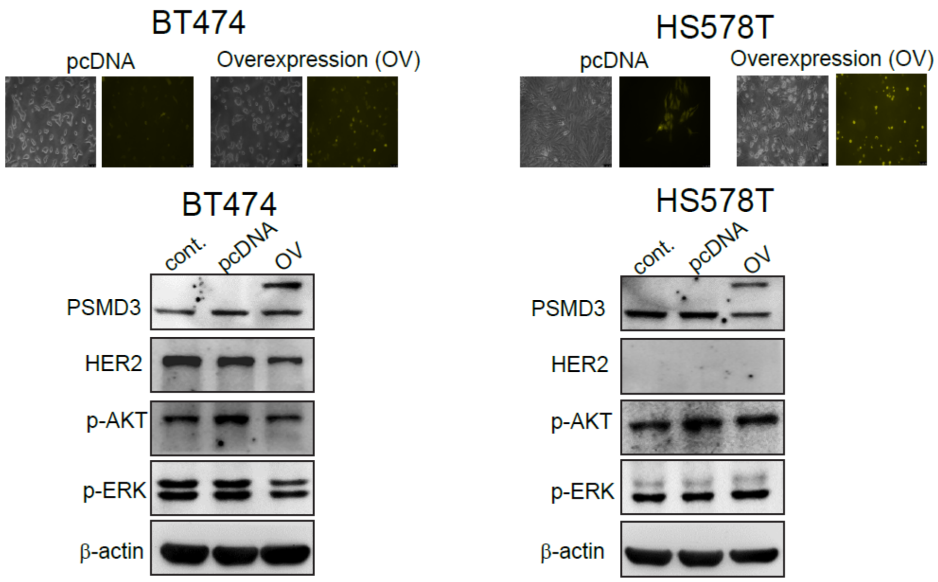

Figure S2. PSMD3 overexpression and its relation to HER2 and HER2 signaling proteins in BT474 and HS578T cell lines.

Table S1. Correlation between PSMD3 H-score and Clinicopathological parameters of breast cancer ($n = 59$).

Parameters	Categories	PSMD3 Low H-Score (n)	PSMD3 High H-Score (n)	p-Value
Age	≤40	7	6	0.332
	>40	30	16	
HER2	Positive	13	8	0.571
	Negative	14	24	
ER	Positive	7	10	0.074
	Negative	30	12	
TNBC	Positive	15	7	0.350
	Non-TNBC	22	15	
Tumor size	T1	2	0	0.726
	T2	20	12	
	T3	3	2	
	T4	1	2	
	* NA=17			
Grade	1	0	3	0.167
	2	13	7	
	3	16	8	
	* NA (12)			
Node	N0	11	9	0.389
	N1	9	3	
	N2	2	4	
	N3	6	4	
	* NA (11)			
Stage	I	3	3	0.819
	II	15	9	
	III	10	8	
	* NA (11)			

ER, Estrogen receptor; HER2, human epidermal growth factor2; TNBC, triple-negative BC; ***NA**, not given. Clinicopathological parameters were assessed using Chi-square analysis. * $p < 0.05$, ** $p < 0.001$. PSMD3 low vs PSMD3 high based on the mean level of PSMD3 H-score.

Table S2. Univariate and multivariate analysis of OS in HER2 positive vs total patients according to PSMD3 H-score.

**Parameters**	**Univariate Analysis (HER2 Positive)**		**Univariate Analysis (Total Patients)**	
	p-value	HR ratio (95% Cl)	*p*-value	HR ratio (95% Cl)
PSMD3 (high vs low)	0.049*	9.072 (0.913–90.190)	0.335	1.991 (0.490–8.086)
Stage (3&4 vs 1&2)	0.556	1.722 (0.282–10.526)	0.608	1.441 (0.358–5.807)
Tumor size (>5 vs ≤5)	0.157	5.857 (0.507–67.617)	0.059	4.386 (0.943–20.395)
Grade (high vs low)	0.486	0.497 (0.070–3.540)	0.481	0.598 (0.143–2.503)
Node (Positive vs negative)	0.656	1.099 (0.490–2.466)	0.150	4.663 (0.573–37.936)
ER(positive vs negative)	0.649	0.607 (0.067–5.486)	0.698	1.374 (0.275–6.864)
**Parameters**	**Univariate analysis (OS)**		**Multivariate analysis (OS)**	
	p-value	HR ratio (95% Cl)	*p*-value	HR ratio (95% Cl)
Stage (3&4 vs 1&2)	<0.001***	1.531 (1.252–1.873)	<0.001***	1.508 (1.214–1.873)
Tumor size (>5 vs ≤5)	0.512	1.1611 (0.743–1.814)	0.466	1.280 (0.660–2.483)
Node (Positive vs negative)	0.013*	1.597 (1.102–2.315)	0.237	1.277 (0.852–1.914)
ER(positive vs negative)	0.448	1.226 (0.724–2.077)	0.206	1.422 (0.824–2.457)
HER2 (positive vs negative)	0.004*	2.139 (1.268–3.609)	0.028*	1.823 (1.067–3.113)
PSMD3 (high vs low)	0.054	1.407 (0.991–1.996)	0.022*	1.518 (1.063–2.169)

CI, confidence interval; ER, Estrogen receptor; HER2, human epidermal growth factor2. *$p < 0.05$. Cox regression analysis, hazard ratio (95% confidence interval).

Table S3. Univariate and multivariate analysis of overall survival (OS) according to TCGA database.

**Parameters**	**Multivariate Analysis (HER2 Positive)**		**Multivariate Analysis (Total Patients)**	
	p-value	HR ratio (95% Cl)	*p*-value	HR ratio (95% Cl)
PSMD3 (high vs low)	0.059	9.934 (0.919–107.373)	0.232	2.556 (0.548–11.918)
Stage (3 vs 1&2)	0.864	0.836 (0.107–6.519)	0.844	0.845 (0.159–4.502)
Tumor size (>5 vs ≤5)	0.744	1.914 (0.039–94.475)	0.107	4.040 (0.739–22.073)
Grade (high vs low)	0.325	3.999 (0.254–63.001)	0.561	0.635 (0.138–2.932)
Node (Positive vs negative)	0.441	0.388 (0.035–4.316)	0.127	5.144 (0.627–42.205)
ER(positive vs negative)	0.604	0.524 (0.046–6.029)	0.490	2.085 (0.259–16.806)

© 2019 by the authors. Licensee MDPI, Basel, Switzerland. This article is an open access article distributed under the terms and conditions of the Creative Commons Attribution (CC BY) license (http://creativecommons.org/licenses/by/4.0/).
